# The effect of bilirubin on the excitability of mitral cells in the olfactory bulb of the rat

**DOI:** 10.1038/srep32872

**Published:** 2016-09-09

**Authors:** Xiao-Juan Chen, Hui-Qun Zhou, Hai-bo Ye, Chun-Yan Li, Wei-Tian Zhang

**Affiliations:** 1 Department of Otorhinolaryngology-Head & Neck Surgery Affiliated Sixth People’s Hospital of Shanghai Jiaotong University, Shanghai, P.R. China

## Abstract

Olfactory dysfunction is a common clinical phenomenon observed in various liver diseases. Previous studies have shown a correlation between smell disorders and bilirubin levels in patients with hepatic diseases. Bilirubin is a well-known neurotoxin; however, its effect on neurons in the main olfactory bulb (MOB), the first relay in the olfactory system, has not been examined. We investigated the effect of bilirubin (>3 μM) on mitral cells (MCs), the principal output neurons of the MOB. Bilirubin increased the frequency of spontaneous firing and the frequency but not the amplitude of spontaneous excitatory postsynaptic currents (sEPSCs). TTX completely blocked sEPSCs in almost all of the cells tested. Bilirubin activity was partially blocked by N-methyl-D-aspartate (NMDA) and α-amino-3-hydroxy-5-methyl-4-isoxazolepro pionic acid (AMPA) receptor antagonists. Furthermore, we found that bilirubin increased the frequency of intrinsic firing independent of synaptic transmission in MCs. Our findings suggest that bilirubin enhances glutamatergic transmission and strengthens intrinsic firing independent of synaptic transmission, all of which cause hyperexcitability in MCs. Our findings provide the basis for further investigation into the mechanisms underlying olfactory dysfunction that are often observed in patients with severe liver disease.

The Beaver Dam Offspring Study found that the overall prevalence of olfactory impairment in the general population was 3.8%^1^. Aging is the leading cause of olfactory decline, and other causes include trauma, neurodegenerative diseases, endocrine changes, vitamin deficiency, and liver disease^2^. Recent studies have investigated the impacts of acute^3^, chronic^4^,^5^, and end-stage liver disease^6^ on the sense of smell. The findings suggest that olfactory function is impaired to varying degrees in patients with liver disease. Dysosmia and hyposmia are common symptoms of hepatitis^5^,^7^. Moreover, the ability to identify odors, but not odor thresholds, is associated with the degree of liver cirrhosis^8^. In patients with liver cirrhosis, it is believed that odors are processed by the central nervous system (CNS) rather than the peripheral^7^ because threshold values reflect peripheral olfactory function, and odor identification is associated with the higher-order processing of olfactory input^9^,^10^,^11^. Studies have shown that olfactory acuity is related to plasma-bilirubin levels in patients with acute hepatitis^3^. Moreover, serum bilirubin levels are correlated with the degree of olfactory dysfunction in patients with liver cirrhosis^12^. Consequently, bilirubin-induced neuronal injury may underlie the olfactory dysfunction in patients with liver disease.

As a neurotoxin, bilirubin can cause multiple neurological deficits. Excitotoxicity, mitochondrial energy failure, and increased intracellular calcium concentration [Ca^2+^]_i_ may be spatially and temporally linked to the molecular pathogenesis of bilirubin-induced neuronal cell injury^13^. Previously, we showed that excitotoxicity was an important contributing factor to bilirubin-induced neuronal injury in the ventral cochlear nucleus and lateral superior olive, auditory brainstem nuclei that are sensitive to bilirubin toxicity^14^,^15^. Pathological changes in the cochlear nucleus and olfactory bulb have been identified in an animal model of kernicterus syndrome^16^. Mitral cells (MCs), the principal cells in the main olfactory bulb (MOB), project to the piriform cortex where olfactory perception occurs^17^. MC excitability is mediated via glutamate acting at the ionotropic glutamate receptor subtypes, α-amino-3-hydroxy-5-methyl-isoxazole-4-propionic acid (AMPA) and *N*-methyl- d-aspartate (NMDA)^18^,^19^,^20^,^21^,^22^. Gamma-aminobutyric acid (GABA), which is released from inhibitory interneurons, periglomerular cells (PGs), and granule cells (GCs), also mediates synaptic transmission in the MOB. The key synaptic transmitters, GABA and glutamate, create an analytical information-processing system in the MOB^23^,^24^,^25^.

We investigated the effect of bilirubin on MC excitability because excitatory output from these cells reflects the encoding of odor information^23^ which may be impaired in patients with hepatic disease owing to high serum bilirubin levels. We used patch-clamp techniques to investigate whether bilirubin induced hyperexcitation in MCs and to examine the underlying mechanisms. We found that bilirubin significantly affected MC excitability, providing direct evidence that high bilirubin levels interfere with MC output and contribute to the olfactory dysfunction associated with liver disease.

## Results

### MC properties

**Figure 1:**
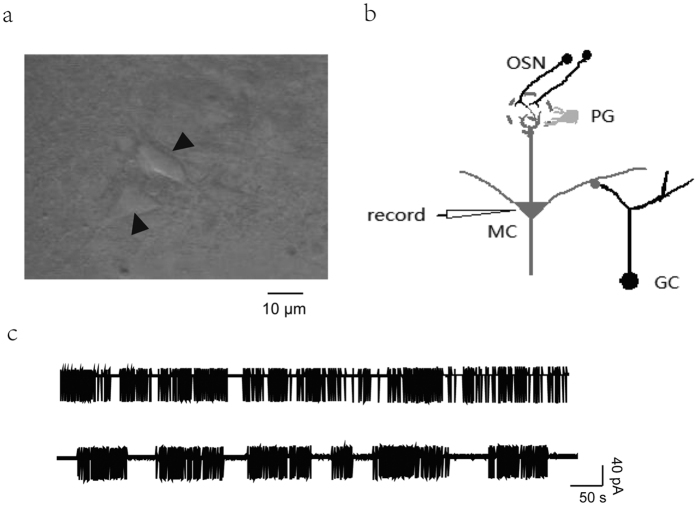
Identification of MCs according to morphological and electrophysiological characteristics. (**a**) Photograph of two typical pyramidal MCs obtained from the MC layer of a (P24) rat. (**b**) Schematic MC recording configuration showing synaptic connections with OSN, PG, and GC. (**c**) MCs exhibited spontaneous firing with repeated bursts separated by short or long pauses. Recordings were made under cell-attached conditions.

In the MOB slices, MCs were identified by their morphology and position within the MC layer. MCs typically have a pyramidal soma that is larger than those of other cell types. The synaptic organization of MCs has been reported. The longest dendrite of MCs is the most extensively apical dendrite which extends to the glomerulus layer, where many of dendrites of PGs are present. GCs contact the secondary dendrites of MCs within the external plexiform layer (Fig. 1)^26^. However, because morphological characteristics alone were not sufficient to identify MCs, electrophysiological characteristics are also examined. We observed firing patterns under cell-attached conditions. All MCs showed burst firing. Some fired in bursts with short pauses between bursts while others had relatively longer pauses between bursts of firing. Figure 1c shows the electrophysiological characteristics of two representative MCs, which are consistent with those previously reported^27^.

### Bilirubin increased the frequency of spontaneous firing in a concentration- dependent manner

**Figure 2:**
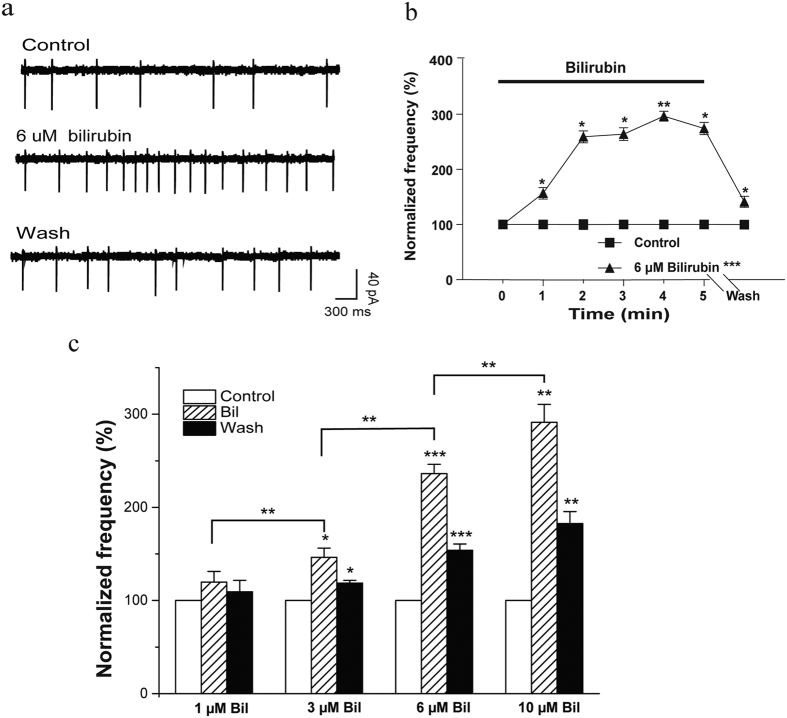
Bilirubin increased the frequency of spontaneous firing in MCs in a concentration-dependent manner. (**a**) Traces of spontaneous firing recorded from a cell before, during, and after the perfusion of 6 μM bilirubin under cell-attached conditions. (**b**) Average spontaneous firing frequency before, during, and after application of bilirubin. Frequencies were averaged every minute and normalized to the mean value during the 5-min control period (n = 6). (**c**) Bar graphs show the average spontaneous firing frequency before, during, and after the application of bilirubin (1, 3, 6, and 10 μM). Vertical error bars represent SE. *p < 0.05; **p < 0.01; ***p < 0.001; ns: no significant difference.

The findings of previous *in vitro* and *in vivo*^28^,^29^ studies suggest that bilirubin- induced CNS injury is the result of its excitotoxic effect. We examined the effect of bilirubin on spontaneous firing in MCs under cell-attached conditions. During the 5-min application period (1, 3, 6, and 10 μM), the MC firing frequency gradually increased and reached a peak value 4–5 min after bilirubin application (Fig. 2a,b). The average normalized spontaneous firing frequencies were 119.7 ± 11% (1 μM: n = 6, ns), 146.3 ± 9% (3 μM: n = 5, *p* < 0.05), 246.1 ± 10% (6 μM: n = 6, *p* < 0.001), and 291.3 ± 19% (10 μM: n = 5, *p* < 0.01) of the control. After the bilirubin was removed by washing with artificial cerebrospinal fluid (ACSF), the firing rate decreased gradually. The firing frequency was 109.9 ± 12% (1 μM: n = 6, ns), 118.6 ± 2% (3 μM: n = 5, *p* < 0.05), 153.9 ± 6% (6 μM: n = 6, *p* < 0.001), and 182.6 ± 12% (10 μM: n = 5, *p* < 0.05) of the control after a 4-min wash. The differences among groups are showed in Fig. 2c. These findings indicate that the bilirubin-induced hyperexcitation and facilitating effect in MCs was concentration-dependent.

### Bilirubin increased the frequency of sEPSCs

**Figure 3:**
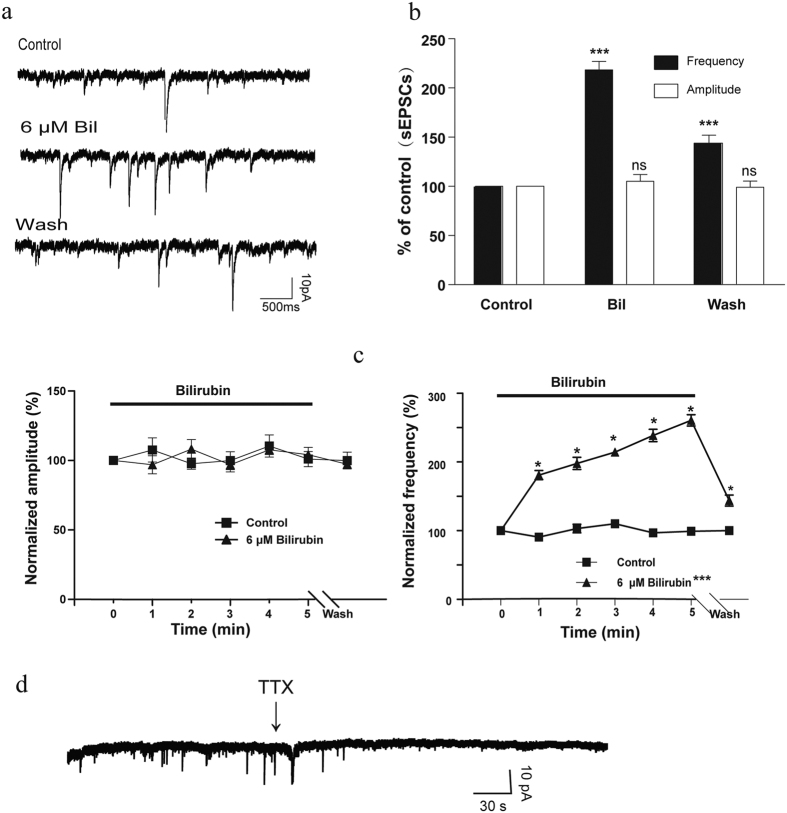
Bilirubin increased the frequency, but not amplitude, of sEPSCs. (**a**) Traces of sEPSCs recorded in a MC before, during, and after the perfusion of 6 μM bilirubin performed in bicuculline and strychnine in voltage clamp mode. (**b**) Bar graphs show the average sEPSC frequency and amplitude of seven neurons before, during (5 min), and after (4 min) the application of bilirubin. (**c**) Average sEPSC amplitude (left) and frequency (right) before, during, and after application of bilirubin (n = 7). Frequency and amplitude of sEPSCs were averaged every minute and normalized to the mean value during the 5-min control period (n = 7). (d) TTX (1 μM) blocked sEPSCs in MC (n = 10). Vertical error bars represent SE. *p < 0.05; ***p < 0.001; ns: no significant difference.

Several factors may alter neuronal excitability, such as changes in synaptic transmission, synaptic connectivity, intrinsic neuronal properties, and intracellular signal transduction. Glutamate, the most prevalent excitatory neurotransmitter in the CNS, can produce long-lasting changes in neuronal excitability. To investigate whether glutamatergic transmission is involved in the bilirubin-induced hyperexcitation of MCs, we recorded sEPSCs in MCs with bicuculline and strychnine to block feedback inhibition from granule and periglomerular cells under voltage-clamp conditions. Because sensory-induced discharge is dependent on multistep excitation rather than direct synaptic connections from the olfactory sensory neurons (OSNs) in MCs^30^, and because MC apical dendrites are relatively longer than those of other neuron types in the MOB^31^, not all MCs have sEPSCs, and their amplitude is relatively small. We were able to record sEPSCs in 7 of 35 MCs. The amplitude and frequency of sEPSCs were 5–20 pA and 2–6 Hz, respectively. The sEPSC frequency increased within 1 min and reached a maximum level about 5 min after bilirubin application (6 μM; Fig. 3a,c). The average sEPSC frequency during the 5-min bilirubin application was 218.1 ± 5% of the control (n = 7, *p* < 0.001). After washing with ACSF for 4 min, the sEPSC frequency decreased to 138.6 ± 2% of the control (n = 7, *p* < 0.001; Fig. 3b). However, bilirubin did not significantly alter the sEPSC amplitude (average: 105.4 ± 3% of the control in the presence of bilirubin, 99 ± 2% of the control after washing for 4 min, n = 7, ns) throughout the bilirubin application period (data shown every minute after bilirubin application; Fig. 3c). These findings suggest that glutamatergic transmission is potentiated by bilirubin in MCs. In the presence of TTX, we found that miniature EPSCs were absent in almost all of the neurons recorded, indicating these events are mainly action potential dependent (Fig. 3d, n = 10).

### Bilirubin-evoked firing of MCs is partially mediated by glutamatergic transmission

**Figure 4:**
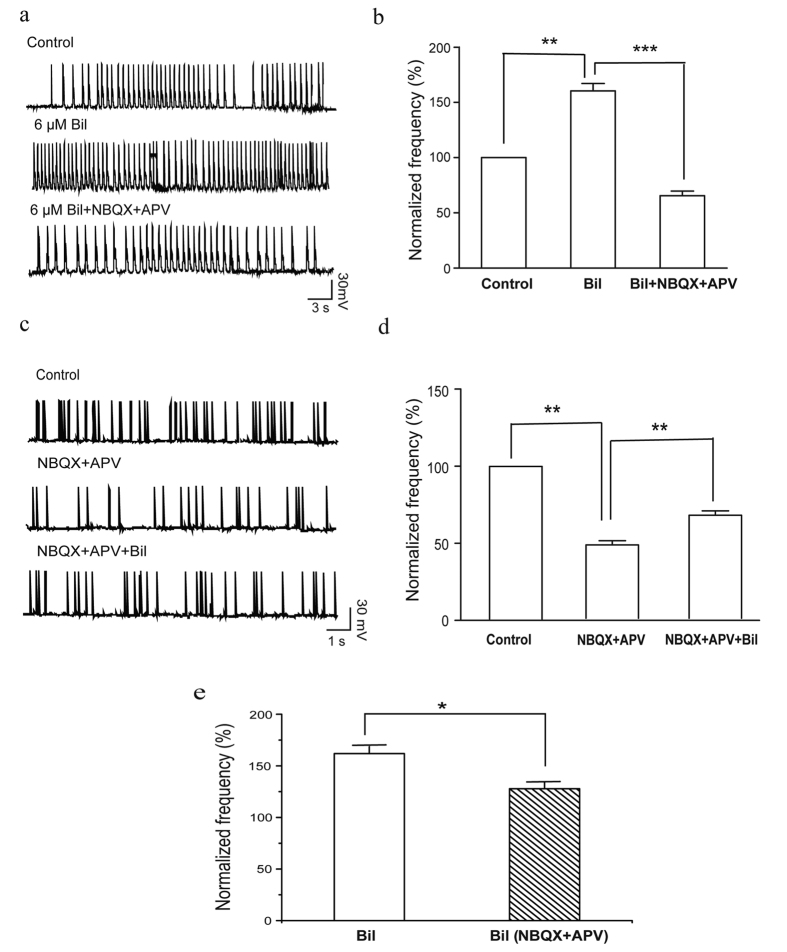
Bilirubin-evoked MC activity was partially mediated by glutamatergic transmission. (**a**) Traces of spontaneous action potentials recorded in a MC before and during the application of bilirubin under whole cell current clamp conditions. The bilirubin-induced increase in firing rate was partially blocked by APV and NBQX. (**b**) Bar graphs show the average spontaneous action potential frequency of MCs (n = 7) before and during the application of bilirubin, and during APV and NBQX co-application with bilirubin. (**c**) Traces of spontaneous firing recorded from a cell before and during the application of APV and NBQX and subsequent co-application of bilirubin with APV and NBQX. (**d**) Bar graphs show the average spontaneous action potential frequency of MCs (n = 5) before and during the application APV and NBQX and co-application of bilirubin with APV and NBQX. (**e**) Bar graphs show the MC firing rate in response to bilirubin alone and bilirubin following pretreatment with APV and NBQX. Vertical error bars represent SE. *p < 0.05; **p < 0.01; ***p < 0.001.

Previously, we showed that bilirubin stimulated glutamate release in lateral superior olive neurons^15^. However, no studies have investigated the role of glutamatergic neurotransmission in bilirubin-induced hyperexcitation in MCs. We found that, under current-clamp conditions, bilirubin perfusion (6 μM) for 5-min markedly increased the MC firing frequency to 160.4 ± 7% of the control (n = 7, *p* < 0.01). Then, we applied AMPA and NMDA receptor antagonists during bilirubin treatment for 5-min. The results showed that the application of 50 μM APV and 10 μM NBQX reduced the firing frequency to 65.6 ± 4% of the control, which was significantly lower than that observed with bilirubin alone (n = 7, *p* < 0.001; Fig. 4a,b). Next, application of the receptor antagonists, 50 μM l–2-amino-5-phosphonovaleri -cacid (APV) and 10 μM 2,3-dihydroxy-6-nitro-7-sulfamoyl-ben-zoquinoxaline -2,3-dione (NBQX) reduced the cell firing frequency to 48.2 ± 3% of the control. The addition of bilirubin to the APV and NBQX solution increased the firing rate to 130.5 ± 6% of the antagonists, which was significantly different from the response to the antagonists alone (n = 5, *p* < 0.001; 6 μM; Fig. 4c,d). Figure 4e shows the effects of bilirubin alone and bilirubin following pretreatment with APV and NBQX. Although bilirubin increased the firing rate in the presence of NBQX and APV, the effect was significantly suppressed compared with that under the bilirubin-alone condition. These results indicate that bilirubin-induced hyperexcitation in MCs is mediated, in part, by glutamatergic neurotransmission.

### Bilirubin increased the intrinsic firing rate of MCs

**Figure 5:**
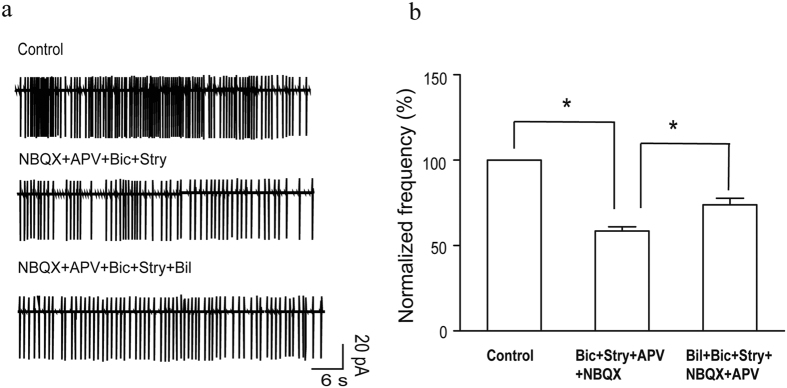
Bilirubin increased synaptic-independent intrinsic firing in MCs. (**a**) Representative recording of intrinsic firing in a MC during the application of the synaptic receptor antagonists APV, NBQX, bicuculline, and strychnine under cell attached conditions. The firing rate increased following the addition of bilirubin. (**b**) Bar graphs show the average spontaneous firing frequency before and during the application of synaptic receptor blockers and during co-application of bilirubin with the synaptic blockers. Vertical error bars represent SE. *p < 0.05.

Intrinsic firing patterns, which generate spontaneous rhythmic firing in the absence of synaptic input, occur in a number of neurons^32^,^33^,^34^,^35^,^36^,^37^,^38^,^39^. This characteristic may contribute to the function of these neurons^40^. These previous findings suggest that intrinsic firing may play a role in the bilirubin-induced hyperexcitation of MCs. To address this issue, we first recorded the spontaneous firing of MCs in the cell-attached configuration and then pharmacologically blocked synaptic-evoked activity using AMPA, NMDA, GABA_A_, and glycine receptor antagonists. We found that the spontaneous firing frequency was reduced to 58.9 ± 3% of the control (n = 5, *p* < 0.05) after the application of 50 μM bicuculline, 3 μM strychnine, 10 μM NBQX, and 50 μM APV (Fig. 5a,b). However, the remaining firing was robust and regular with bursts, just as before the synaptic blockade (Fig. 5a). These findings are similar to those of a previous study^41^. Interestingly, despite the blockade of all synaptic transmission, the cell firing rate increased to 125.1 ±  4% of all blockers during the 5-min bilirubin application, which was significantly different from the results under the blockers-alone condition (n = 5, *p* < 0.05; Fig. 5b).

## Discussion

Excitotoxicity is a generally accepted consequence of bilirubin-induced hyperexcitation^13^, which is caused by the free fraction of unconjugated bilirubin that is not bound to plasma proteins and can freely enter the brain^42^. The excitotoxic effects of bilirubin have been documented in various CNS areas, including the ventral cochlear nuclei, subthalamic nucleus, and hippocampus^13^. The central processing of odors is disrupted by elevated serum bilirubin levels. Therefore, we hypothesized that the administration of bilirubin would significantly interfere with the activity of MCs, the principal output neurons of the MOB, and their role in encoding odor information. MCs were identified using morphological and electrophysiological criteria, which were consistent with those described previously. We recorded spontaneous firing in MCs using the cell-attached patch-clamp technique. We found that bilirubin at a concentration of 1 μM did not significantly increase the MC firing rate, while at concentrations of 3, 6, and 10 μM, bilirubin significantly increased the frequency of spontaneous firing in a concentration-dependent manner, indicating that bilirubin induced hyperexcitation in MOB neurons at concentrations comparable to those of hyperbilirubinemia. Amin *et al*.^43^ reported similar findings in auditory brainstem nuclei. The MC firing rate is associated with the encoding of odor information^23^. Therefore, our finding that bilirubin concentrations exceeding 3 μM changed MC activity suggests a possible mechanism for odor dysfunction in patients with liver disease.

Glutamate is the primary excitatory neurotransmitter in the CNS^28^,^30^. Glutamate excitotoxicity is hypothesized to damage neurons via excessive synaptic release of glutamate and consequent overstimulation of glutamate receptors^44^. We previously showed that bilirubin stimulated presynaptic glutamate release, causing increased activity in lateral superior olive neurons, which is consistent with bilirubin-induced glutamate excitotoxicity^15^. The excitatory activity in the MCs is mediated by glutamate via ionotropic glutamate AMPA, and NMDA receptors^45^. Therefore, we tested the hypothesis that glutamate and glutamate receptors are involved in the bilirubin-induced hyperexcitation of MCs. We found that bilirubin increased the frequency but not the amplitude of glutamatergic sEPSCs. These findings suggest that bilirubin can potentiate glutamatergic synaptic transmission without influencing the sensitivity of postsynaptic glutamate receptors. Glutamatergic EPSCs can originate either olfactory nerve axons or the dendrodendritic release of glutamate from other mitral/tufted cells. To clarify whether the action of bilirubin depends on action-potential, 1 μM TTX was added to ACSF. This showed that sEPSCs were blocked completely in almost all of the cells tested. Furthermore, the bilirubin-induced neuronal hyperexcitation was not completely blocked by glutamate receptor antagonists in our study (Fig. 4a,b), implying that the increase in glutamatergic neurotransmission is not the only mediator of bilirubin-induced hyperexcitation in MCs. It is possible that bilirubin also increases firing by acting on the ion channels in the soma of mitral/tufted cells leading to increase in firing and subsequent increase in release of glutamate from their dendrites. Our findings support the notion that enhanced glutamatergic neurotransmission is associated with the abnormal coding of olfactory information^46^, which may occur in patients with hepatic encephalopathy, a severe liver disease^47^.

Intrinsic firing independent of synaptic transmission has been detected in neurons in several areas of the CNS. This phenomenon depends on intrinsic membrane properties and has been shown to play an essential role in the refinement of neural connections, particularly during development^8^,^48^,^49^. To determine whether MCs exhibited intrinsic firing patterns and whether they were affected by bilirubin, we recorded neuronal firing in the presence of excitatory and inhibitory synaptic receptor inhibitors. We found that the MCs exhibited intrinsic rhythmic firing. Moreover, bilirubin increased the frequency of intrinsic firing. Balu *et al*.^50^ hypothesized that the intrinsic membrane properties that drive intermittent spike clusters in MCs are important for the precise timing of the spikes evoked by odors for the encoding of olfactory information. MC firing activity is perceived as encoded odor messages. Thus, our finding that bilirubin significantly increased the frequency of intrinsic firing in MCs suggests that it may play a key role in olfactory function as a biochemical modulator. Intrinsic firing in MCs is controlled by several ion channels (e.g., HCN, 4-AP-sensitive K-currents, *I*_D_-like currents, and TTX-sensitive Na-current)^50^. The exact biophysical mechanisms involved in intrinsic MC activity are not known. Moreover, those ion channels controlling intrinsic MC firing that are affected by bilirubin remain to be identified.

In conclusion, our findings show that bilirubin facilitates glutamatergic synaptic transmission and intrinsic firing in MCs, which may contribute to bilirubin-induced hyperexcitation. Our results suggest a possible cellular mechanism for explaining the loss of the ability to identify odors experienced by patients with liver disease. Furthermore, our findings provide the basis for further research into the prevention and treatment of olfactory dysfunction in patients with liver disease.

## Materials and Methods

Our study was approved by the Ethics Review Committee for Animal Experimentation at Shanghai Jiao Tong University. The experiments were performed in accordance with the Guiding Principles for the Care and Use of Animals, and all efforts were made to minimize the possible pain and discomfort of the animals during the experiments.

### Preparation of MOB slices

Extraction and dissection of the olfactory bulbs were performed as described previously^51^,^52^. Postnatal 21–28-day-old Sprague–Dawley rats were anesthetized with sodium pentobarbital (55 mg/kg, intraperitoneal) and then decapitated. The MOB was quickly removed from the skull and placed into oxygenated ice-cold cutting solution containing (in mM): 83 NaCl, 2.5 KCl, 0.5 CaCl_2_, 3.3 MgSO_4_, 26.2 NaHCO_3_, 1 NaH_2_PO_4_, 22 D-glucose, and 72 sucrose. Transverse slices were cut at a thickness of 300 μm using a microslicer (VT-1000S; Leica, Nussloch, Germany). Slices were transferred to ACSF containing the following (in mM): 124 NaCl, 5 KCl, 1.2 KH_2_PO_4_, 1.3 MgSO_4_, 2.4 CaCl_2_, 24 NaHCO_3_, and 10 glucose with an osmolarity of 300 mOsm and a pH of 7.3 when bubbled with 95% O_2_–5% CO_2_ at 37 °C for at least 30 min. The slices were then moved to a recording chamber at room temperature (24–26 °C) before recording.

### Reagents

The reagents used in the experiments included free bilirubin, bicuculline, strychnine, NBQX, and APV and TTX (all from Sigma, St. Louis, MO, USA). As a stock solution, bilirubin was dissolved in 0.1 M NaOH at 1 mM, stored in single-use aliquots in the dark at −20 °C (<48 h), and diluted to the final solution concentration before use. The bilirubin solution was protected from light at all times. Bicuculline and APV were prepared as stock solutions using 100% dimethyl sulfoxide (DMSO; Sigma) and diluted to the required concentration in ACSF immediately before use, resulting in a maximum DMSO concentration of <0.1%. Other drugs were prepared as stock solutions in distilled water and diluted to the required concentrations in ACSF immediately before use. All drugs were applied locally via a square glass capillary (0.4 mm in width, Cat. #64-0121, Warner Instrument, New Haven, CT, USA), which was placed on the slice as close to the MC as possible.

### Electrophysiological measurements and data analyses

Cell-attached and whole-cell recordings were performed using a patch-clamp amplifier (EPC10; HEKA, Elektronik, Lambrecht, Germany). The electrode capacitance and liquid-junction potential were compensated. Data were filtered at 1–3 kHz and sampled at 3–10 kHz using a Dell computer equipped with PATCHMASTER software (HEKA, Elektronik). The patch pipettes were pulled from borosilicate capillary glass through two stages using a vertical pipette puller (P-9; Narishige, Tokyo, Japan). The resistance of the electrode was 3–7 MΩ. Patch electrodes were filled with the following solutions for cell-attached recordings (in mM): 97.5 K-gluconate, 32.5 KCl, 0.5 ethyleneglycol-bis(2-aminoethyl)-N,N,N′,N′,-tetraacetic acid (EGTA), 40 4-(2-hydroxyethyl)-1-piperazineethanesulfonic acid (HEPES), and 1 MgCl_2_, 290–300 mOsm, pH 7.3; they were filled with the following solutions for whole-cell recordings (in mM): 120 K-gluconate, 5 KCl, 2 MgCl_2_, 0.05 EGTA, 10 HEPES, 2 Mg-ATP, 0.4 Mg-GTP, 10 creatine phosphate, at 290–300 mOsm and pH 7.3. Some of the recordings used to assess spontaneous excitatory postsynaptic currents (sEPSCs) in MCs were performed using low Cl^−^based with cesium in the pipette solution (in mM): 127 cesium methanesulfonate, 20 NaCl, 20 HEPES, 0.4 EGTA, 5 TEA-Cl, 3 QX314-Cl, 2.5 MgATP and 0.33 NaGTP at 300–310 mOsm, and pH 7.3. We conducted one recording per brain slice. All experiments were performed at room temperature (24–26 °C). Differences in the frequency of action potential currents and sEPSCs were analyzed, using Wilcoxon signed-ranks tests for differences in the frequency of action potential currents and sEPSCs intra-group comparisons, using Mann-Whitney *U*-test for independent concentration groups comparisons. The Statistical Package for the Social Sciences version 17.0 (SPSS, Chicago, IL, USA) was used to conduct the statistical tests. The results are expressed as means  ±  standard errors (SEs), and *p*-values <0.05 were deemed to indicate statistical significance.

## Additional Information

**How to cite this article**: Chen, X.-J. *et al*. The effect of bilirubin on the excitability of mitral cells in the olfactory bulb of the rat. *Sci. Rep*. **6**, 32872; doi: 10.1038/srep32872 (2016).
